# LOGGIC/FIREFLY-2: a phase 3, randomized trial of tovorafenib vs. chemotherapy in pediatric and young adult patients with newly diagnosed low-grade glioma harboring an activating *RAF* alteration

**DOI:** 10.1186/s12885-024-11820-x

**Published:** 2024-01-30

**Authors:** Cornelis M. van Tilburg, Lindsay B. Kilburn, Sébastien Perreault, Rene Schmidt, Amedeo A. Azizi, Ofelia Cruz-Martínez, Michal Zápotocký, Katrin Scheinemann, Antoinette Y. N. Schouten-van Meeteren, Astrid Sehested, Enrico Opocher, Pablo Hernáiz Driever, Shivaram Avula, David S. Ziegler, David Capper, Arend Koch, Felix Sahm, Jiaheng Qiu, Li-Pen Tsao, Samuel C. Blackman, Peter Manley, Till Milde, Ruth Witt, David T. W. Jones, Darren Hargrave, Olaf Witt

**Affiliations:** 1 Hopp Children’s Cancer Center Heidelberg (KiTZ), Heidelberg, Germany; 2 Clinical Cooperation Unit Pediatric Oncology, German Cancer Research Center (DKFZ), Heidelberg, Germany; 3 Department of Pediatric Oncology, Hematology, Immunology and Pulmonology, Heidelberg University Hospital, Heidelberg, Germany; 4 German Cancer Consortium (DKTK), Heidelberg, Germany; 5 National Center for Tumor Diseases (NCT), Heidelberg, Germany; 6 Children’s National Hospital, Washington, DC USA; 7 CHU Sainte-Justine, Université de Montréal, Montréal, QC Canada; 8 Institute of Biostatistics and Clinical Research, Münster, Germany; 9 Department of Pediatrics and Adolescent Medicine, Comprehensive Center for Pediatrics and Comprehensive Cancer Center, Medical University of Vienna, Vienna, Austria; 10 Neuro-oncology Unit, Pediatric Cancer Center, Hospital Sant Joan de Déu, Barcelona, Spain; 11 Department of Paediatric Haematology and Oncology, Charles University, Second Faculty of Medicine and University Hospital Motol, Prague, Czech Republic; 12 Division of Oncology-Hematology, Children’s Hospital of Eastern Switzerland, St. Gallen, Switzerland; 13 Faculty of Health Sciences and Medicine, University of Lucerne, Lucerne, Switzerland; 14 Department of Pediatrics, McMaster Children’s Hospital and McMaster University, Hamilton, Canada; 15 Department of Neuro-oncology, Princess Máxima Center for Pediatric Oncology, Utrecht, The Netherlands; 16 Department of Pediatrics and Adolescent Medicine, Rigshospitalet, Copenhagen, Denmark; 17 Pediatric Hematology, Oncology and Stem Cell Transplant Division, Padua University Hospital, Padua, Italy; 18 German HIT-LOGGIC-Registry for LGG in Children and Adolescents, Charité-Universitätsmedizin Berlin, Corporate Member of Freie Universität Berlin and Humboldt-Universität zu Berlin, Berlin, Germany; 19 Department of Radiology, Alder Hey Children’s Hospital NHS Foundation Trust, Liverpool, UK; 20 Kids Cancer Centre, Sydney Children’s Hospital, Randwick, NSW Australia; 21 Lowy Cancer Research Centre, Children’s Cancer Institute, University of New South Wales, Sydney, NSW Australia; 22 School of Clinical Medicine, University of New South Wales, Sydney, NSW Australia; 23 Department of Neuropathology, Charité - Universitätsmedizin Berlin, Berlin, Germany; 24 DKTK Partner Site, Berlin, Germany; 25 Department of Neuropathology, German Cancer Research Center (DKFZ), University Hospital Heidelberg and CCU Neuropathology, German Consortium for Translational Cancer Research (DKTK), Heidelberg, Germany; 26 Day One Biopharmaceuticals, Brisbane, CA USA; 27 Division of Pediatric Glioma Research, German Cancer Research Center (DKFZ), Heidelberg, Germany; 28 UCL Great Ormond Street Institute of Child Health and Great Ormond Street Hospital for Children, London, UK

**Keywords:** Chemotherapy, First-line, Pediatric low-grade glioma, pLGG, Tovorafenib, Child, BRAF, MAPK

## Abstract

**Background:**

Pediatric low-grade glioma (pLGG) is essentially a single pathway disease, with most tumors driven by genomic alterations affecting the mitogen-activated protein kinase/ERK (MAPK) pathway, predominantly *KIAA1549::BRAF* fusions and BRAF V600E mutations. This makes pLGG an ideal candidate for MAPK pathway-targeted treatments. The type I BRAF inhibitor, dabrafenib, in combination with the MEK inhibitor, trametinib, has been approved by the United States Food and Drug Administration for the systemic treatment of BRAF V600E-mutated pLGG. However, this combination is not approved for the treatment of patients with tumors harboring *BRAF* fusions as type I RAF inhibitors are ineffective in this setting and may paradoxically enhance tumor growth. The type II RAF inhibitor, tovorafenib (formerly DAY101, TAK-580, MLN2480), has shown promising activity and good tolerability in patients with *BRAF-*altered pLGG in the phase 2 FIREFLY-1 study, with an objective response rate (ORR) per Response Assessment in Neuro-Oncology high-grade glioma (RANO-HGG) criteria of 67%. Tumor response was independent of histologic subtype, *BRAF* alteration type (fusion vs. mutation), number of prior lines of therapy, and prior MAPK-pathway inhibitor use.

**Methods:**

LOGGIC/FIREFLY-2 is a two-arm, randomized, open-label, multicenter, global, phase 3 trial to evaluate the efficacy, safety, and tolerability of tovorafenib monotherapy vs. current standard of care (SoC) chemotherapy in patients < 25 years of age with pLGG harboring an activating *RAF* alteration who require first-line systemic therapy. Patients are randomized 1:1 to either tovorafenib, administered once weekly at 420 mg/m^2^ (not to exceed 600 mg), or investigator’s choice of prespecified SoC chemotherapy regimens. The primary objective is to compare ORR between the two treatment arms, as assessed by independent review per RANO-LGG criteria. Secondary objectives include comparisons of progression-free survival, duration of response, safety, neurologic function, and clinical benefit rate.

**Discussion:**

The promising tovorafenib activity data, CNS-penetration properties, strong scientific rationale combined with the manageable tolerability and safety profile seen in patients with pLGG led to the SIOPe-BTG-LGG working group to nominate tovorafenib for comparison with SoC chemotherapy in this first-line phase 3 trial. The efficacy, safety, and functional response data generated from the trial may define a new SoC treatment for newly diagnosed pLGG.

**Trial registration:**

ClinicalTrials.gov: NCT05566795. Registered on October 4, 2022.

**Supplementary Information:**

The online version contains supplementary material available at 10.1186/s12885-024-11820-x.

## Background

Pediatric low-grade glioma (pLGG) is a heterogeneous group of World Health Organization (WHO) grade 1 and 2 tumors comprising several subgroups and is the most frequent pediatric central nervous system (CNS) tumor diagnosis, with 1200 to 1500 new cases per year in the United States (US) [1]. Although pLGG is a low-grade tumor with an excellent 10-year overall survival (OS) rate of 94%, the 10-year progression-free survival (PFS) rate in case of an indication for systemic treatment with standard of care (SoC) chemotherapy is only 44% [2, 3]. Therefore, pLGG may be considered a chronic disease in patients whose tumors are unresectable or cannot be completely resected, who will often require several treatment lines throughout life [4]. The late adverse effects of the disease and treatment of it in combination with the damage to important functional structures puts a heavy burden on patients and can lead to loss of visual function and impairment of neurologic, endocrine, and cognitive functioning [5–7].

The current SoC first-line systemic treatment is chemotherapy, most commonly a combination of vincristine and carboplatin (V/C). Although the regimens used by the European Society for Paediatric Oncology—Brain Tumour Group—low-grade glioma working group (SIOPe-BTG LGG WG) and the Children’s Oncology Group (COG) differ slightly, both regimens show a similar outcome [8, 9]. The SIOPe-BTG LGG WG reported a response rate of 29% (24 weeks after treatment start) and a five-year PFS rate of 45% [9], whereas the COG reported a response rate of 35% (end of treatment) and a five-year event-free survival rate of 39% [8]. A single-arm phase 2 study of single-agent vinblastine (VBL) resulted in a 19% response rate and a five-year PFS rate of 53% [10]. Several countries/institutions now utilize VBL as first-line therapy on the basis of this trial.

Treatment with V/C or VBL may be accompanied by significant adverse effects, including bone marrow toxicity, neurotoxicity, hearing loss, renal dysfunction, and allergic reactions [3]. The burden of toxicity, frequent hospital visits as well as a significant number of patients progressing after first-line treatment, motivated the search for alternative strategies. In addition, these chemotherapy regimens appear even less effective in infants with pLGG [11], underscoring the need for novel treatment options in this particular subgroup of patients with poor prognosis [9].

Pediatric low-grade glioma is predominantly a single pathway disease driven by alterations in the mitogen-activated protein kinase (MAPK) signaling pathway (also known as the RAS-RAF-MEK-ERK pathway). *KIAA1549::BRAF* fusions, BRAF V600E mutations, *FGFR1* alterations and loss of function mutations of the neurofibromin 1 (*NF1*) gene are the most frequent molecular alterations [12–18]. In addition, oncogene-induced senescence and the senescence-associated secretory phenotype have recently been reported to play an important role in pLGG [19, 20].

As a single pathway disease, pLGG is an ideal candidate for the development of targeted treatments. In a phase 2 trial comparing the combination of the type I BRAF inhibitor dabrafenib and the MEK inhibitor trametinib to chemotherapy in patients with BRAF V600E-mutated pLGG requiring first-line systemic treatment, an objective response rate (ORR) of 47% and a median PFS of 20.1 months were observed, whereas SoC chemotherapy treatment with V/C resulted in an ORR of 11% and a median of PFS 7.4 months [21]. Subsequently, the US Food and Drug Administration (FDA) approved dabrafenib combined with trametinib as a systemic treatment for BRAF V600E-mutated pLGG [22]. The use of type I BRAF inhibitors is limited to patients with tumors harboring a BRAF V600E mutation due to the risk of paradoxical activation of the MAPK pathway and accelerated tumor growth if used in patients with tumors harboring a *RAF* fusion, such as those involving *BRAF* or *CRAF/RAF1* [23]. In recurrent or progressive disease, MEK inhibitors, such as selumetinib and trametinib, have shown activity [24–26]. However, data generated from ongoing studies of various MAPK pathway inhibitors have shown that responses in pLGGs driven by either BRAF V600E mutations (treated with type I RAF inhibitors and/or MEK inhibitors) or *KIAA1549::BRAF* fusions (treated with MEK inhibitors) are often only durable for as long as the drug can be administered [27, 28]. Furthermore, while the ORRs of MEK inhibitors are encouraging, responses can be relatively slow [25], and current MEK inhibitors are poorly brain-penetrant and associated with significant peripheral toxicities, mostly dermatological, but also cardiac and ophthalmological adverse events (AEs) [29, 30]. Thus, high target selectivity, CNS penetration properties, short time to response, and favorable tolerability over a long course of treatment are key determinants of sustained clinical activity and therefore treatment success [26].

Tovorafenib (formerly DAY101, TAK-580, MLN2480) is an investigational, oral, brain-penetrant, selective, small molecule, type II RAF inhibitor. In contrast to the approved type I RAF inhibitors, tovorafenib inhibits both wild-type BRAF and CRAF/RAF1 kinases and, importantly, hyperactivated signaling resulting from *BRAF* fusions, including the *KIAA1549::BRAF* fusion [31].

Tovorafenib was shown to inhibit the kinase activity of BRAF kinase domain fusions with various 5′ gene partners, most notably fusion with the *KIAA1549* gene. In cellular assays, tovorafenib inhibited *KIAA1549::BRAF* fusion kinase activity with comparable potency to inhibition of BRAF V600E and without the paradoxical activation of the MAPK pathway reported for type I BRAF inhibitors [32]. Tovorafenib blocked downstream pERK signaling and had less severe dermatological, cardiac, or ophthalmological toxicities compared to other RAF or MEK inhibitors [33–35]. Finally, tovorafenib had greater CNS penetration compared with the type I RAF inhibitor dabrafenib [32]. The clinical activity of tovorafenib in the currently ongoing phase 2 FIREFLY-1 (PNOC026) study in pediatric patients with *BRAF*-altered, recurrent, or progressive pLGG harboring a *BRAF* fusion or BRAF V600E mutation was recently reported. In the registrational arm (69 evaluable patients), the ORR primary endpoint as determined by Response Assessment in Neuro-Oncology high-grade glioma (RANO-HGG) criteria per independent radiology review committee (IRC) was 67% [33, 35]. Responses were observed in patients with tumors harboring BRAF V600E mutations who had received prior MAPK-targeted therapy. Finally, the median time to response was 2.8 months according to RANO-HGG criteria [33, 35], which is shorter in comparison to MEK inhibitors [25] and may have beneficial impact on the functional outcomes of patients.

The promising tovorafenib activity, manageable safety profile, oral availability, once weekly (QW) dosing as well as strong scientific rationale are the basis for the ongoing LOGGIC/FIREFLY-2 trial. The purpose of this trial is to evaluate the efficacy, safety, and tolerability of oral tovorafenib monotherapy versus SoC intravenous chemotherapy in patients with pLGG harboring an activating *RAF* alteration requiring first-line systemic therapy. The primary efficacy endpoint, ORR, will be evaluated for tovorafenib versus SoC chemotherapy as determined by an IRC using RANO-LGG criteria [36]. In addition, PFS will be a key secondary endpoint tested in a hierarchical manner following ORR for the final assessment of efficacy. Importantly, this study also includes endpoints to evaluate improvements in neurologic outcomes, visual function in patients with optic pathway glioma (OPG), and patient-reported outcomes to better describe the overall impact on patients’ lives and activities of daily living. Lastly, independent of this trial, tumor material will be submitted to the molecular platform LOGGIC Core BioClinical Data Bank [37], by sites participating in this parallel study, to explore the identification of prognostic and predictive molecular biomarkers including the recently described MAPK inhibitor sensitivity score [38].

## Methods/design

### Study design

**Fig. 1:**
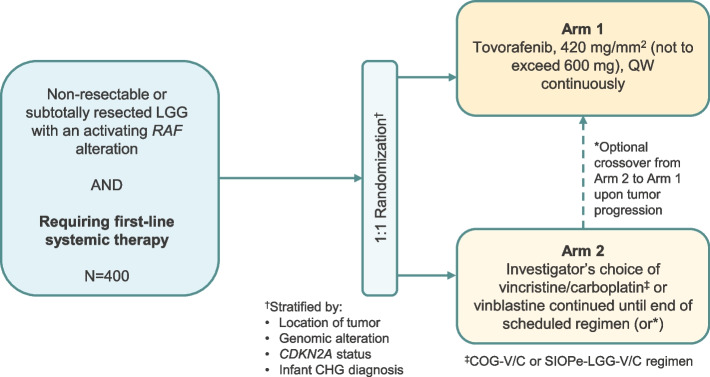
Design of the LOGICC/FIREFLY-2 (NCT05566795) trial. The study consists of a screening phase, a treatment phase, an end of treatment visit, a 30-day safety follow-up visit, and a long-term follow-up period. The total length of the study, from screening through to the end of the study is expected to be seven years. CHG: Chiasmatic-hypothalamic glioma; COG-V/C: Children’s Oncology Group-vincristine/carboplatin; LGG: Low-grade glioma; QW: Once weekly; SIOPe-LGG-V/C: Society of Paediatric Oncology Europe-low-grade glioma vincristine/carboplatin

LOGGIC/FIREFLY-2 (NCT05566795) is a two-arm, randomized, open-label, multicenter, global, phase 3 trial to evaluate the efficacy, safety, and tolerability of tovorafenib monotherapy vs. SoC chemotherapy in patients with pLGG harboring an activating *RAF* alteration who require first-line systemic therapy. Approximately 400 treatment-naïve patients will be randomized at a ratio of 1:1 to either Arm 1 (tovorafenib) or Arm 2 (investigator’s choice of SoC chemotherapy) (Fig. 1). SoC chemotherapy will consist of either COG-V/C [39], SIOPe-LGG-V/C [9] or VBL [10].

Prior to any study treatment administration, patients fulfilling all eligibility criteria will be centrally randomized to a treatment arm using the IRT (interactive response technology). Randomization between treatment arms will be stratified by primary location of the tumor (supratentorial midline vs. other), type of genomic *RAF* alteration (fusion vs. mutation), *CDKN2A* status (deletion vs. wild-type/unknown), and infant chiasmatic-hypothalamic glioma (CHG) diagnosis (yes vs. no). Patients who experience progressive disease in Arm 2 can crossover to Arm 1 and receive tovorafenib. No allocation concealment will be necessary as this is an open-label study.

### Study objectives

#### Primary objective

The primary objective is to compare the ORR of tovorafenib monotherapy (Arm 1) vs. investigator’s choice of SoC chemotherapy (Arm 2) as assessed by the IRC per RANO-LGG [36] criteria in pediatric and young adult patients with LGG harboring an activating *RAF* alteration who require first-line systemic therapy. The IRC will review all images and response assessments will be determined by the IRC.

#### Key secondary objectives

- Comparison of PFS between study arms as assessed by the IRC per RANO-LGG criteria
- Comparison of duration of response (DoR) between study arms as assessed by the IRC per RANO-LGG criteria
- Comparison of OS between study arms
- Comparison of safety and tolerability between study arms by assessment of AEs, serious AEs, treatment-emergent AEs, laboratory values, and vital signs

#### Other secondary objectives

- Comparison of neurologic function in the following domains between study arms: motor function, daily living skills, communication, and socialization using the Vineland Adaptive Behavior Composite Scales (VABS)
- Comparison of changes in visual acuity outcomes between study arms in patients with OPG
- Comparison of ORR between study arms as assessed by the IRC per RANO-HGG [40] criteria and by Response Assessment in Pediatric Neuro-Oncology for LGG (RAPNO-LGG) [41] criteria
- Comparison of clinical benefit rate (CBR) between study arms as assessed by the IRC per RANO-LGG, RANO-HGG, and RAPNO-LGG criteria
- Comparison of time to response (TTR) between study arms as assessed by the IRC per RANO-LGG, RANO-HGG, and RAPNO-LGG criteria
- Comparison of PFS between study arms as assessed by the IRC per RANO-HGG and RAPNO-LGG criteria
- Comparison of DoR between study arms as assessed by the IRC per RANO-HGG and RAPNO-LGG criteria

#### Exploratory objectives

- Comparison of ORR, CBR, TTR, PFS, and DoR between study arms as assessed by investigators per RANO-LGG criteria
- Comparison of changes in growth and development of patients between study arms
- Comparison of chemotherapy-induced peripheral neuropathy outcomes between study arms by pediatric-modified total neuropathy score in patients ≥ five years of age
- Comparison of neuro-endocrine morbidity between study arms
- Comparison of changes in total tumor volume following treatment between study arms
- Comparison of changes in apparent diffusion coefficients within the tumor following treatment between study arms using diffusion-weighted imaging analysis based on MRI scan data
- Patient-reported outcomes (PRO) assessments: comparison of changes in quality of life (QoL) and health utilities measures between study arms using the Pediatrics Quality of Life™-Core Module (PedsQL-Core), Pediatrics Quality of Life™-Cancer (PedsQL-Cancer), and Patient-Reported Outcomes Measurement Information System (PROMIS^®^) assessment
- Comparison of time to initiation of next treatment following discontinuation of primary therapy between study arms
- Comparison of cystic involution (the change in total tumor volume including possible cystic parts) between study arms measured using MRI and assessed by the IRC
- Comparison of the efficacy and safety of individual SoC chemotherapy regimens vs. tovorafenib
- Determination of the ORR and disease control rate of patients who begin tovorafenib after discontinuing SoC chemotherapy due to radiographic progression as assessed by the IRC per RANO-LGG, RANO-HGG, and RAPNO-LGG criteria, and by the investigators per RANO-LGG criteria
- Evaluation of the concordance of prior local laboratory *RAF* molecular profiling with a central *RAF* alteration assay being evaluated by the sponsor (Day One Biopharmaceuticals)
- Explore whether early responses in infant CHG at six and 12 weeks correlate with response after 24 weeks of treatment
- Assessment of the pharmacokinetics (PK) of tovorafenib through blood sample collection from patients randomized to the tovorafenib arm (Arm 1)

### Study population

The phase 3 LOGGIC/FIREFLY-2 in front-line pLGG is enrolling globally and the study is ongoing; the first patient was dosed in March 2023 [42]. The accrual period is estimated to be ~ two years to achieve a total of 400 evaluable patients pooled over both arms [43]. The trial is being conducted at academic centers with patients being recruited from ~ 100 sites, including the SIOPe LOGGIC (Low Grade Glioma in Children) consortium in Europe, the Asia–Pacific region (Singapore, South Korea, Australia and New Zealand), and North America (Canada and the US). Patients are eligible for inclusion in the study if they are < 25 years of age with a histopathological diagnosis of LGG or neuroepithelial tumor (grade 1–2 per the 2021 WHO classification for CNS; [44]), harboring a known activating *RAF* alteration as identified through molecular assays routinely performed at Clinical Laboratory Improvement Amendments (CLIA) of 1988 or other similarly certified laboratories. Patients must be evaluable by RANO-LGG, that is, they must have at least one measurable lesion imaged up to 28 days prior to treatment initiation that can be defined by T2/FLAIR and reproducibly measured by MRI in at least two-dimensions of at least 10 mm in size, and visible on two or more axial slices that are preferably ≤ 5 mm apart with 0 mm skip. Additionally, an indication for first-line systemic therapy is required, meaning that the tumor is non-resectable (either completely unresectable or partially resected with residual tumor that can no longer be resected) at the time of enrollment, and the patient qualifies for one of the following tumor-related indications for first-line drug treatment:

- At primary diagnosis:
  - ∘ Present with CHG and be < one year of age at diagnosis, independent of neurologic and/or visual symptoms
  - ∘ Diencephalic syndrome
  - ∘ Patients with OPG meeting visual-related criteria
  - ∘ Neurologic symptoms/deficits
- After completion of an initial observation phase (so called “watch and wait” strategy):
  - ∘ Manifestation of diencephalic syndrome
  - ∘ Patients with OPG exhibiting progression of visual impairment
  - ∘ Deterioration of neurologic symptoms
  - ∘ Radiologic progression (local investigator judgement without pre-specification)

Patients < 16 years of age and patients ≥ 16 years of age, will be required to exhibit Lansky and Karnofsky performance status scores, respectively, of ≥ 70%. Furthermore, for children and adolescents, their parents/legal guardians must agree to comply with study procedures, including treatment, laboratory monitoring, and required clinic visits for the duration of their participation in the study.

Key exclusion criteria include diagnosis of a patient with any of the following tumor histological types: schwannoma, subependymal giant cell astrocytoma (tuberous sclerosis), or diffuse intrinsic pontine glioma, even if histologically diagnosed as WHO grade 1–2. Patients with *NF1*-driven tumors are excluded. Furthermore, patients with a tumor exhibiting activating molecular alterations in addition to activating *RAF* alterations are also ineligible for participation even if the tumors are histologically of a low-grade. Activating molecular alterations leading to exclusion include, but are not limited to:

- *IDH1/2* mutations
- Histone H3 mutations
- *NF1* loss-of-function mutations
- *MYBL* alterations
- *FGFR* mutations or fusions
- Diagnosis, or suspected diagnosis of patients with neurofibromatosis type 1 or 2 via genetic testing or current diagnostic clinical criteria would also lead to exclusion.

A full list of inclusion and exclusion criteria are included in Additional File 1.

### Arms and interventions

#### Arm 1

Patients randomized to Arm 1 receive tovorafenib at a dose of 420 mg/m^2^ (not to exceed 600 mg), in the form of a tablet or liquid suspension, QW, continuously on days 1, 8, 15, and 22 of 28-day (four week) cycles. Dosage is adjusted for body surface area (BSA) calculated per the Mösteller Formula [√((height × weight)/3600)]. Patients with a BSA of 0.6 m^2^ or less will be required to receive the liquid suspension formulation. If appropriate in the opinion of the investigator, patients with a BSA of 0.7 m^2^ or greater may change formulations at any point during treatment. Administration of tovorafenib will continue until radiographic progression based on RANO-LGG criteria as determined by the investigator and confirmed by the IRC, unacceptable toxicity, or the patient withdraws consent (for treatment schema, see Additional File 2). Patients may continue on tovorafenib even following radiographic progression at the investigator’s discretion if there is evidence that the patient is still deriving clinical benefit.

#### Arm 2

Patients randomized to Arm 2 are treated with one of three SoC chemotherapy regimens based on the investigator’s choice: COG-V/C regimen, SIOPe-LGG-V/C regimen, or VBL regimen [9, 11, 39]. The SoC chemotherapy regimen administered to each patient is determined prior to randomization (for treatment schema, see Additional File 2). Patients randomized to an Arm 2, receiving the COG-V/C regimen are treated with vincristine (BSA ≥ 0.6 mg/m^2^: 1.5 mg/m^2^/day without exceeding 2 mg/day; BSA < 0.6 mg/m^2^: 0.05 mg/kg/day to 0.80 mg/day) administered intravenously (IV) QW through weeks 1–10 of a 12-week induction period; and carboplatin (BSA ≥ 0.6 mg/m^2^: 175 mg/m^2^/day; BSA < 0.6 mg/m^2^: 175 mg/m^2^ to 90 mg) administered IV once during weeks 1, 2, 3, 4, 7, 8, 9, and 10. Patients do not receive any treatment for the last two weeks of the induction period. During the maintenance period (eight six-weekly cycles from week 12 to completion of treatment at 60 weeks), vincristine is administered on weeks 1, 2, and 3 and carboplatin on weeks 1, 2, 3, and 4.

Patients randomized to Arm 2 receiving the SIOPe-LGG-V/C regimen undergo an induction period of 24 weeks (7 cycles), during which they receive vincristine (body weight ≥ 10 kg: 1.5 mg/m^2^/day without exceeding 2 mg/day; body weight < 10 kg: 0.05 mg/kg/day; age < six months: further 1/3 dose reduction) IV QW from weeks 1–10, and on weeks 13, 17, and 21. During this period, carboplatin (body weight ≥ 10 kg: 550 mg/m^2^/day, without exceeding 1050 mg; body weight < 10 kg: 18.3 mg/kg/day; age < six months: further 1/3 dose reduction) is administered as a one-hour infusion IV on weeks 1, 4, 7, 10, 13, 17, and 21. Following a four-week period where patients do not receive any treatment, they then enter a consolidation phase until completion of treatment at 81 weeks, during which they receive vincristine and carboplatin in six-week cycles, with vincristine administered on weeks 1, 2, and 3; and carboplatin on week 1.

The Arm 2 VBL regimen comprises VBL (BSA ≥ 0.6 m^2^: 6 mg/m^2^/day, without exceeding 10 mg; BSA < 0.6 m^2^: BSA dose × 1/30 × body weight [kg]/day) administered IV QW in four-week cycles until completion of treatment at 70 weeks.

For patients experiencing treatment delays or disruptions, investigators may extend treatment with SoC chemotherapies for up to two additional cycles. Other than the end of study, treatment may be discontinued if a patient exhibits radiographic progression based on RANO-LGG criteria as determined by the investigator and confirmed by the IRC, unacceptable toxicity, or the patient withdraws consent. Patients in Arm 2 who demonstrate radiographic progression during the treatment phase or after completion of chemotherapy may be eligible to receive tovorafenib (Fig. 1).

#### Dose modifications

If patients in Arm 1 experience an AE that is clinically or medically intolerable based on evaluation by National Cancer Institute Common Terminology Criteria for Adverse Events Version 5.0 (which include AEs that have previously been observed with either tovorafenib administration or other therapies that have a similar mechanism of action), tovorafenib dosing will be interrupted until resolution to grade 1 or the AE reverts to a baseline level. Upon resolution and/or reversion, dosing may be restarted at the same dose or a lower dose, at the discretion of the local investigator. If the dose is reduced, re-escalation to a higher dose may be permitted after approval by the sponsor’s medical monitor or designee. The initial starting dose (420 mg/m^2^ [not to exceed a dose of 600 mg]) is based on BSA. The reconstituted liquid formulation (25 mg/mL) is required for a BSA 0.3 to 0.6 m^2^. For tablets (100 mg) and a BSA 0.9 ≥ 1.9 m^2^, the first dose reduction is 100 mg from starting dose; the second dose reduction is 100 mg from the first dose reduction (i.e., 200 mg total from the initial starting dose). For the liquid formulation, dose reductions vary from 1 mL (BSAs of 0.3 and 0.5 m^2^), 2 mL (BSAs of 0.4, 0.6–0.8 m^2^), 3 mL (BSAs of 0.9–1.2 m^2^) and 4 mL (BSAs of 1.3 ≥ 1.9 m^2^) for the first dose reduction from the starting dose and 1 mL (BSAs of 0.3 and 0.4 m^2^), 2 mL (BSAs of 0.5–0.9 m^2^), 3 mL (BSAs of 1.0–1.3 m^2^) and 4 mL (BSAs of 1.4 ≥ 1.9 m^2^) for the second dose reduction from the first dose reduction (i.e., between 2 mL [BSA 0.3 m^2^] to 8 mL [BSAs of 1.4 ≥ 1.9 m^2^] total from the initial starting dose). Chemotherapy dosing for patients in Arm 2 may be reduced or temporarily delayed due to toxicity in accordance with protocol-defined criteria; re-escalation to a higher dose following resolution or reversion of the AE to baseline may be permitted only after approval by the sponsor’s medical monitor or designee. All patients on either treatment arm who experience drug-related toxicity requiring a recovery period longer than 42 days will be withdrawn from study drug administration unless, for patients in Arm 1 receiving tovorafenib, there is evidence of clinical benefit, and no alternative treatment is available as determined by the investigator and approved by the sponsor’s medical monitor or designee.

### Tests and evaluations

#### Physical evaluations

All radiographic tumor measurements will be carried out using MRI of the brain and/or spine. This will occur up to 28 days prior to starting treatment, and approximately every 12 weeks throughout the treatment and long-term follow-up (LTFU) periods. Neurologic function measured by VABS (motor function, daily living skills, communication and socialization) will be evaluated one, two, and five years after treatment initiation. In addition, screening for visual acuity is required for all patients; baseline assessments will be taken during screening. For patients in Arm 1 (and Arm 2 cross-over patients receiving tovorafenib) with deficiencies in visual function related to OPG, visual acuity testing involving a fundus examination with comment on retinal abnormalities and optic disc, and if possible, visual fields to confrontation, and best corrected visual acuity (BCVA) via logarithm of the minimum angle of resolution assessment will be performed at every radiographic response assessment, the end-of-treatment visit, and every six months during the LTFU period. For all other patients in Arm 1 and all patients in Arm 2, fundus examinations with a comment on retinal abnormalities and BCVA testing will be completed as needed during patient visits.

In addition to standard ophthalmology examination, monitoring for safety will include physical examination, neurologic examination, dermatology examination, cardiac evaluation, bone assessment (for patients with Tanner stage < 4–5), Karnofsky/Lansky performance score, clinical AEs, laboratory variables and vital signs, including height and weight.

#### QoL evaluations

PedsQL-Core, PedsQL-Cancer and PROMIS^®^ are being assessed at Screening, every 52 weeks from week 5 to end of treatment, and at year 5 as part of the LTFU. Arm 2 has an additional assessment at year 2 in the LTFU. This difference is because Arm 1 involves treating to progression. All PROs are administered according to the scale developer's intended age group. For children under four years of age, caregiver(s)/parent(s) will complete the proxy-reporting PROs. For children above five years of age, both the child and parent will complete self-report and parent proxy report version of the scale, if applicable. PROs will only be conducted if local language translation is available.

#### PK evaluations

The dose of tovorafenib will be taken in the clinic on PK sampling days to ensure that PK samples are collected at scheduled times. The schedule of blood sample collections is cycle 1 day 1, cycle 2 day 1 (± three-day window), and cycle 4 day 1 (± three-day window): pre-dose and two hours post-dose, and then every subsequent third cycle through to cycle 13 day 1 (± three-day window): pre-dose, and in the event of toxicity. If a patient receiving tovorafenib experiences an AE that fits the criteria of a severe adverse event (SAE) as determined by the investigator, a blood sample will be collected, when clinically feasible, for measurement of drug concentrations at or around that time.

#### Statistical analysis

To assess the ORR per the primary endpoint, the planned sample size of approximately 400 patients will provide around 85% power to detect a 15% improvement for the tovorafenib arm at a two-tailed level of significance of 0.05, assuming a 30% ORR in the control arm and a dropout rate of up to 10%. To assess PFS as per the key secondary endpoint, the planned sample size of approximately 400 patients will provide around 85% power to detect a hazard ratio of 0.67 for PFS at a two-tailed level of significance of 0.05, assuming the median PFS for the tovorafenib arm is 4.5 years versus a median of three years for the control arm. The ORR by RANO-LGG (IRC-assessed) primary analysis is expected to occur ~ 12 months after the last patient is randomized; a PFS analysis will occur later.

For statistical hypothesis testing of efficacy endpoints, a multiple testing procedure will be applied to control the overall type I error rate at a two-sided alpha level of 5% for the hypotheses testing of the primary (ORR per RANO-LGG criteria) and the key secondary endpoints (PFS per RANO-LGG criteria, DoR per RANO-LGG criteria). Analysis of the primary endpoint ORR as assessed by the IRC per RANO-LGG criteria will be done first at a two-sided alpha level of 5%. If this test meets the pre-specified boundary for statistical significance, then the key secondary endpoints will be tested. It should be noted that the statistical analysis plan for the LOGGIC/FIREFLY-2 trial may be subject to change based on emerging data from FIREFLY-1 trial.

#### Data monitoring

To ensure a high level of safety monitoring during this trial, an independent Data Monitoring Committee (DMC) has been established and will meet periodically to review safety and efficacy data across the entire tovorafenib program. The purpose of the DMC will be to ensure the ethical conduct of the LOGGIC/FIREFLY-2 study and to protect the safety interests of patients in this study. Based on its review, the DMC will provide the Sponsor with recommendations regarding trial modification, continuation, or termination. Roles and responsibilities of DMC and Sponsor as well as meeting schedule and format of information are set forth in a charter.

## Discussion

Patients with pLGG are in need of alternative, effective treatments with less toxicity, deeper and more durable activity and importantly, better neurologic, visual, and patient-reported outcomes. In the registrational arm (Arm 1) of the phase 2 FIREFLY-1 trial evaluating tovorafenib in pediatric patients with pLGG, rapid responses were observed with a median time to response of 2.8 months (RANO-HGG), 5.5 months (RAPNO-LGG [pending adjudication]) and 4.2 months (RANO-LGG), as of a December 22, 2022 data cutoff. Overall response rates of 67% (RANO-HGG, confirmed complete response [cCR] or partial response [PR], includes three unconfirmed partial responses [uPRs]), 51% (RAPNO-LGG, cCR, PR or minor response [MR] [pending adjudication], includes four uPR and four unconfirmed MR [uMR]), and 49% (RANO-LGG, cCR, PR or MR, includes eight uPR and two uMR) were observed with a clinical benefit rate of 93% (RANO-HGG, cCR, PR or stable disease [SD]), 87% (RAPNO-LGG, cCR, PR, MR or SD [pending adjudication]) and 83% (RANO-LGG, cCR, PR, MD or SD) [33]. No difference in response rate was noted for patients that had previously been treated with MAPK pathway inhibitors. Furthermore, of 136 patients treated in Arms 1 and 2 (safety analysis set), only five discontinued treatment due to AEs (of those, four were treatment related); 39 required dose reductions or treatment interruptions due to treatment-related AEs [33, 35].

The promising phase 2 tovorafenib activity data in combination with the tolerability and safety of this oral monotherapy in patients with pLGG led to the decision of the SIOPe-BTG LGG WG to nominate tovorafenib for a comparison with SoC chemotherapy in the first-line phase 3 trial. This resulted in a collaboration with Day One Biopharmaceuticals who became the sponsor of the LOGGIC/FIREFLY-2 trial. Pediatric oncology drug development is hampered by many hurdles and challenges, especially in pediatric neuro-oncology, and the collaboration between an academic group and a biopharmaceutical company as in the LOGGIC/FIREFLY-2 trial accelerates accessibility of innovative drugs for children with cancer and at the same time provides opportunities for the scientific advancement of the field [45].

In the context of pLGG being a chronic disease, in addition to radiologic responses, functional endpoints such as neurologic outcomes, visual function in OPG, and patient-reported outcomes are of extreme importance and will be assessed throughout this study. As this is a registrational trial, the primary efficacy endpoint in this study is based on RANO-LGG criteria, the same criteria used in registrational study CDRB436G2201 (NCT02684058) of dabrafenib in combination with trametinib in pediatric patients with pLGG harboring a BRAF V600 mutation [36, 46]. Radiologic response will also be measured by RAPNO-LGG criteria [41] as a secondary endpoint; data from this trial will contribute towards the clinical validation of the existing RAPNO working group consensus recommendations.

Although not in the study protocol, tissue can be sent from participating sites for molecular profiling via the LOGGIC Core BioClinical Databank [37]. This will not only confirm the mandatory activating *RAF* alterations but will also allow for exploration and correlation of complex biomarkers based on RNA sequence analysis and clinical outcomes. To this aim, a novel MAPK inhibitor sensitivity score was recently developed to guide biomarker driven future trials, such as those investigating relapse and/or combination therapy [38].

The ongoing LOGGIC/FIREFLY-2 trial will determine how the promising phase 2 activity data in combination with the tolerability and safety of tovorafenib will translate when compared with SoC chemotherapy in the first-line treatment setting [47]. Compared with most currently applied chemotherapy regimens, which require in-clinic intravenous treatment, patients randomized to tovorafenib will receive an oral drug that is taken at home once weekly, that is available in both tablet and a pediatric friendly oral suspension that can be also given via a nasogastric or gastric tube, allowing continuation of daily activities such as attending school. For future patients, the efficacy and safety data generated from this study can potentially address the unmet clinical need in the treatment of pLGG and aims at defining the new SoC treatment for this disease in a global effort.

### Supplementary Information

**Additional file 1**.

**Additional file 2**.

## Data Availability

Data sharing is not applicable to this article as no datasets have been generated or analyzed. However, in the future, relevant data supporting the findings of the study will be shared in the context of publication(s). To request individual participant data associated with any Day One Biopharmaceuticals clinical trial, please email clinical@dayonebio.com.

## Abbreviations

AEs: Adverse events
BCVA: Best corrected visual acuity
BSA: Body surface area
CBR: Clinical benefit rate
cCR: Confirmed complete response
CHG: Chiasmatic-hypothalamic glioma
CLIA: Clinical Laboratory Improvement Amendments
CNS: Central nervous system
COG-V/C: Children’s Oncology Group-vincristine/carboplatin
DMC: Data monitoring committee
DoR: Duration of response
FLAIR: Fluid attenuated inversion recovery
HGG: High-grade glioma
IRC: Independent radiology review committee
IV: Intravenously
LGG: Low-grade glioma
LOGGIC: Low-Grade Glioma in Children
LTFU: Long-term follow up
MAPK: Mitogen-activated protein kinase
MR: Minor response
MRI: Magnetic resonance imaging
ORR: Objective response rate
OPG: Optic pathway glioma
OS: Overall survival
PFS: Progression-free survival
pLGG: Pediatric low-grade glioma
PRO: Patient-reported outcomes
QW: Once weekly
RANO: Response Assessment in Neuro-Oncology
RAPNO: Response Assessment in Pediatric Neuro-Oncology
SAE: Severe adverse event
SD: Stable disease
SIOPe-BTG-LGG WG: European Society for Paediatric Oncology Brain Tumour Group—low-grade glioma working group
SIOPe-LGG-V/C: Society of Paediatric Oncology Europe-low-grade glioma vincristine/carboplatin
SoC: Standard of care
TTR: Time to response
VABS: Vineland Adaptive Behavior Composite Scales
uMR: Unconfirmed minor response
uPR: Unconfirmed partial response
VBL: Vinblastine
WHO: World Health Organization
