# Supplementary material for: LOGGIC/FIREFLY-2: a phase 3, randomized trial of tovorafenib vs. chemotherapy in pediatric and young adult patients with newly diagnosed low-grade glioma harboring an activating RAF alteration
Source: BMC Cancer. 2024 Jan 30;24:147. doi: 10.1186/s12885-024-11820-x (PMC10826080; doi:10.1186/s12885-024-11820-x)
Supplement: Supplementary file 1 — Additional file 1. [file 12885_2024_11820_MOESM1_ESM.docx]

**Additional file 1**

**Selection of Study Population**

Potential patients and/or their parents must sign an informed consent form (ICF) and pediatric assent form, where applicable, before any study-specific Screening tests may be conducted.

**Inclusion Criteria:**

Patients must meet all of the following criteria to be eligible for enrollment in the study:

1. Patient is less than 25 years of age with a low-grade glioma harboring a documented known activating RAF alteration, as identified through molecular assays performed at CLIA or other similarly certified laboratories.

2. Histopathological diagnosis of glioma or glioneuronal tumor (Grade 1 or 2, according to 2021 WHO Classification for CNS tumors).

3. Confirmation that sufficient FFPE material and/or fresh frozen tumor tissue are available. If not available, a fresh biopsy should be performed.

4. At least one measurable T2/FLAIR lesion that can be reproducibly measured in at least two dimensions of at least 10 mm, visible on two or more axial slices that are preferably, at most, 5 mm apart with 0-mm skip). Imaging must be performed within 28 days of the initiation of treatment.

5. In case of direct postoperative enrollment (including stereotactic biopsy), postoperative MRI must be performed within 72 hours following surgery.

6. Indication for first-line systemic therapy:

Tumor is non-resectable* AND patient qualifies for one of the following tumor-related indications for first-line drug treatment:

At diagnosis:

a) All infants with chiasmatic-hypothalamic glioma (infant CHG) below one year of age at diagnosis independent from neurologic and/or visual symptoms, or

b) Diencephalic syndrome, or

c) Visual-related criteria (OPG patients only), or

d) Neurologic symptoms/deficits, or

After initial observation phase:

e) Manifestation of diencephalic syndrome, or

f) Visual progression (OPG patients only), or

g) Deterioration of neurologic symptoms, or

h) Radiologic progression.

*Non-resectable refers to the tumor status at time of enrollment (ie, either completely unresectable or sub-totally resected with residual tumor that can no longer be resected).

7. Patients must have adequate hematologic function, as defined by the following:

- ANC ≥1000/mm^3^, and
- Platelet count ≥75.0 × 109/L (transfusions allowed per institutional guidelines; last transfusion >2 weeks prior to start of treatment), and
- Hemoglobin ≥10.0 g/dL (transfusions allowed per institutional guidelines; last transfusion >2 weeks prior to start of treatment).

8. Adequate liver function defined as:

- Total bilirubin ≤1.5 × upper limit of normal (ULN) for age; patients with documented Gilbert’s Disease may be enrolled with Sponsor approval provided total bilirubin ≤2.0 × ULN, and
- Aspartate aminotransferase (AST) and alanine aminotransferase (ALT) ≤2.5 × ULN.

9. Adequate cardiac function defined as:

- Left ventricular ejection fraction (LVEF) ≥50% as measured by echocardiography (ECHO) or multiple-gated acquisition (MUGA) scan, or fractional shortening (FS) ≥25% as measured by ECHO, within 28 days before start of treatment (while not receiving medications for cardiac function), and
- No history or current diagnosis of cardiac disease indicating significant risk of safety (uncontrolled or significant cardiac disease).

10. Adequate renal function defined as: Serum creatinine within normal limits (as defined below), or estimated glomerular filtration rate (eGFR) ≥60 mL/min/1.73 m^2^ based on local institutional practice for determination.

| **Age (at Screening)** | **Maximum Serum Creatinine (mg/dL), Conventional Unit** | **Maximum Serum Creatinine (μmol/L), SI Unit** |
| --- | --- | --- |
| <2 years of age | 0.36 | 31.82 |
| 2 to <5 years of age | 0.43 | 38.01 |
| 5 to <12 years of age | 0.61 | 53.93 |
| 12 to <15 years of age | 0.81 | 71.61 |
| 15 to <19 years of age (male) | 1.08 | 95.47 |
| 15 to <19 years of age (female) | 0.84 | 74.26 |
|  | | |

11. Thyroid function tests must be consistent with stable thyroid function per Investigator. Patients on a stable dose of thyroid replacement therapy for a minimum of 1 week before start of treatment are eligible.

12. Lansky (those younger than 16 years of age) or Karnofsky (those 16 years of age or older) Performance Status ≥70%. Patients who are unable to walk because of paralysis, but who can sit in a wheelchair, will be considered ambulatory for the purpose of assessing the performance score.

13. Male and female patients of reproductive potential must agree to use double, highly effective birth control methods for the full duration of study treatment and for an additional 180 or 120 days following the last dose of tovorafenib or chemotherapy, respectively.

14. Patients must have recovered from any prior surgery.

15. Patients receiving steroids for tumor associated symptoms must be on a stable dose (eg, no initial/loading dose or no increase) for 14 days prior to start of treatment.

16. Patients must be able to swallow tablets or liquid, or administer through gastric access via a nasal or gastric tube.

17. Parent/guardian of child or adolescent patient must have the ability to understand, agree to, and sign the study ICF and applicable pediatric assent form before initiation of any protocol-related procedures, including data and tumor material transfer, must be provided according to International Council for Harmonisation (ICH)/ Good Clinical Practice (GCP) and national/local regulations.

18. Patients and parent(s)/legal guardian must agree to comply with study treatment, laboratory monitoring, and required clinic visits for the duration of study participation.

**Exclusion Criteria**

Patients meeting any of the following criteria are to be excluded from the study:

1. Patient has any of the following tumor-histological findings:

a) Schwannoma

b) Subependymal giant cell astrocytoma (tuberous sclerosis)

c) Diffuse intrinsic pontine glioma, even if histologically diagnosed as WHO grade 1–2

2. Patient’s tumor has additional activating molecular alterations (even if histologically low grade) including, but not limited to any of the following:

a) *IDH1/2* mutation

b) Histone H3 mutation

c) Fibroblast growth factor receptor (*FGFR*) mutations or fusions

d)  *MYBL* alterations

e) *NF1* loss-of-function mutation

3. Known or suspected diagnosis of neurofibromatosis type 1 or 2 (NF-1/NF-2) via genetic testing or current diagnostic clinical criteria.

4. Prior or ongoing nonsurgical anticancer therapy for this indication (eg, chemotherapy, oral/IV targeted therapy) including radiation.

5. History of any major disease, other than the diagnosis of low-grade glioma, that might interfere with safe protocol participation.

6. Major surgery within 14 days (two weeks) prior to start of treatment (does not include surgical resection of the tumor, central venous access, cyst fenestration or cyst drainage, or ventriculoperitoneal shunt placement or revision).

7. Patient has an active systemic bacterial, viral, or fungal infection.

8. Patients who are neurologically unstable despite adequate treatment (eg, uncontrolled seizures).

9. History of drug reaction with eosinophilia and systemic symptoms (DRESS) syndrome, Stevens Johnsons syndrome (SJS), or hypersensitivity to any drug with similar chemical structure or to any other excipient present in the pharmaceutical form of the investigational medicinal products.

10. Patient has clinically significant active cardiovascular disease, or history of myocardial infarction, cerebrovascular event (stroke), or deep vein thrombosis/pulmonary embolism within 6 months prior to start of treatment, ongoing cardiomyopathy, or current prolonged QT interval corrected for heart rate by Fridericia’s formula (QTcF) interval >470 milliseconds based on pretreatment electrocardiogram (ECG).

11. Any psychological, familial, sociological, or geographical condition potentially hampering compliance with the study protocol and follow-up schedule; those conditions should be discussed with the patient before inclusion in the trial.

12. Current treatment with a strong cytochrome P450 2C8 (CYP2C8) or 3A4 (CYP3A4) inhibitor/inducer other than those allowed. Medications that are substrates of CYP2C8 or CYP3A4 are allowed but should be used with caution.

13. Current use of a prohibited medical or herbal preparation or requires any of these medications during the study, including any anticancer therapy.

14. Patient is pregnant, lactating, or expecting to become pregnant during study participation.

15. Patient has National Cancer Institute (NCI) CTCAE v5.0 Grade ≥2 nausea and vomiting, malabsorption requiring supplementation, or significant bowel or stomach resection or chronic medical condition that would preclude adequate absorption of tovorafenib.

16. Patient has CTCAE v5.0 Grade ≥3, CPK elevation (>5 × ULN – 10 × ULN).

17. Current participation in other interventional clinical trials or observation period of competing trials, respectively. Concurrent observational or bio-sampling studies may be allowed with approval from Sponsor. Any prior investigational agent must have undergone a washout period of at least four weeks or five half-lives from start of treatment, whichever is greater.

18. Other unspecified reasons that, in the opinion of the Investigator, make the patient unsuitable for enrollment.
