# Supplementary material for: LOGGIC/FIREFLY-2: a phase 3, randomized trial of tovorafenib vs. chemotherapy in pediatric and young adult patients with newly diagnosed low-grade glioma harboring an activating RAF alteration
Source: BMC Cancer. 2024 Jan 30;24:147. doi: 10.1186/s12885-024-11820-x (PMC10826080; doi:10.1186/s12885-024-11820-x)
Supplement: Supplementary file 2 — Additional file 2. [file 12885_2024_11820_MOESM2_ESM.pptx]

## Slide 1
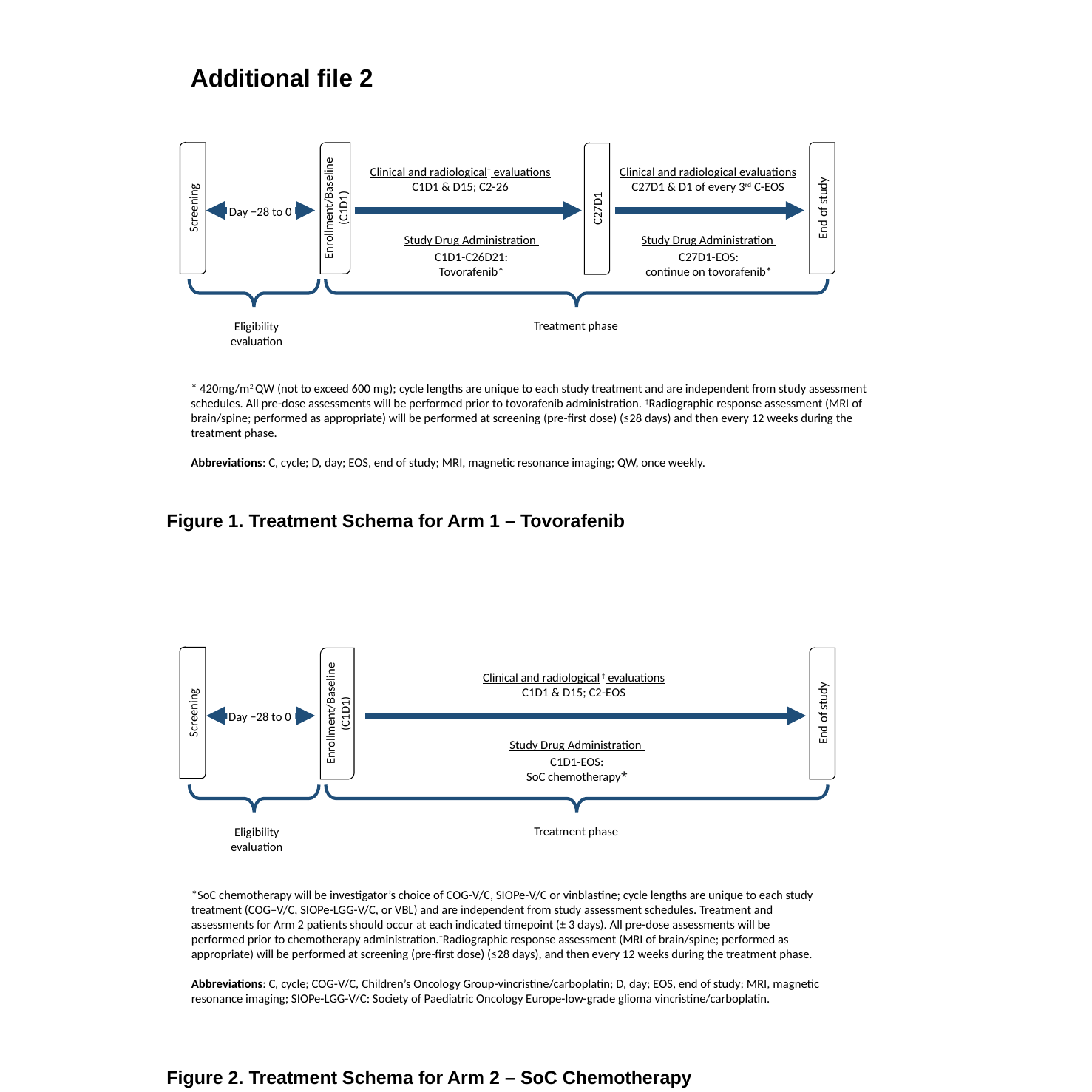

Additional file 2
End of study
Screening
Enrollment/Baseline (C1D1)
C27D1
Clinical and radiological† evaluations
C1D1 & D15; C2-26
Study Drug Administration
C1D1-C26D21:
Tovorafenib*
Treatment phase
Eligibility evaluation
Clinical and radiological evaluations
C27D1 & D1 of every 3rd C-EOS
Day −28 to 0
Study Drug Administration
C27D1-EOS:
continue on tovorafenib*
* 420mg/m2 QW (not to exceed 600 mg); cycle lengths are unique to each study treatment and are independent from study assessment schedules. All pre-dose assessments will be performed prior to tovorafenib administration. †Radiographic response assessment (MRI of brain/spine; performed as appropriate) will be performed at screening (pre-first dose) (≤28 days) and then every 12 weeks during the treatment phase.
Abbreviations: C, cycle; D, day; EOS, end of study; MRI, magnetic resonance imaging; QW, once weekly.
Figure 1. Treatment Schema for Arm 1 – Tovorafenib
Screening
End of study
Enrollment/Baseline (C1D1)
Clinical and radiological † evaluations
C1D1 & D15; C2-EOS
Study Drug Administration
C1D1-EOS:
SoC chemotherapy*
Treatment phase
Eligibility evaluation
Day −28 to 0
*SoC chemotherapy will be investigator’s choice of COG-V/C, SIOPe-V/C or vinblastine; cycle lengths are unique to each study treatment (COG–V/C, SIOPe-LGG-V/C, or VBL) and are independent from study assessment schedules. Treatment and assessments for Arm 2 patients should occur at each indicated timepoint (± 3 days). All pre-dose assessments will be performed prior to chemotherapy administration.†Radiographic response assessment (MRI of brain/spine; performed as appropriate) will be performed at screening (pre-first dose) (≤28 days), and then every 12 weeks during the treatment phase.
Abbreviations: C, cycle; COG-V/C, Children’s Oncology Group-vincristine/carboplatin; D, day; EOS, end of study; MRI, magnetic resonance imaging; SIOPe-LGG-V/C: Society of Paediatric Oncology Europe-low-grade glioma vincristine/carboplatin.
Figure 2. Treatment Schema for Arm 2 – SoC Chemotherapy
